# Effects of transport stress on physiological responses and milk production in lactating dairy cows

**DOI:** 10.5713/ajas.18.0108

**Published:** 2018-08-27

**Authors:** Heeok Hong, Eunchae Lee, In Hyung Lee, Sang-Rak Lee

**Affiliations:** 1 Department of Medical Science, Konkuk University School of Medicine, Seoul 05029, Korea; 2 Department of Animal Bioscience and Technology, Konkuk University, Seoul 05029, Korea

**Keywords:** Transport Stress, Physiological Response, Hematological Responses, Milk Production

## Abstract

**Objective:**

This study was conducted to investigate the effect of transport stress on physiological and hematological responses and milk performance in lactating dairy cows.

**Methods:**

Ten lactating dairy cows were randomly divided into 2 groups. The treatment group (TG) was transported 200 km for 4 h by truck, and the control group (NTG) was restrained by stanchion for 4 h in Konkuk University farm. Blood and milk samples were collected at 24 h pre-transport; 1, 2, and 4 h during transport; and 2, 24, and 48 h post-transport. Milk yields were measured at 24 h pre-transport, 0 h during transport, and 24, 48, and 72 h post-transport.

**Results:**

Leukocyte, neutrophil, and monocyte numbers in the TG were significantly higher than those of the NTG at each experimental time point. Lymphocyte numbers in the TG were significantly (p<0.05) higher than those of the NTG at 48 h post-transport. Additionally, the neutrophil:lymphocyte ratio of the TG was 45% and 46% higher than that of the NTG at 4 h during transport and 2 h post-transport, respectively. There were no significant differences in erythrocyte numbers, hemoglobin concentrations, platelet numbers, and hematocrit percentages between two groups. Cortisol levels in the TG were significantly (p<0.05) higher than those in the NTG. Milk yields in the TG were lower than those in the NTG. The somatic cell count (SCC) of the TG was significantly (p<0.05) higher than that of the NTG at 1 and 2 h during transport; that of the TG increased dramatically at 1 h during transport and gradually decreased subsequently.

**Conclusion:**

Transport stress increased blood parameters including leucocyte, neutrophil, and monocyte numbers by increased cortisol levels, but did not affect erythrocytes, hemoglobin and hematocrit levels. Additionally, transport resulted in a decrease in milk yield and reduced milk quality owing to an increase in milk SCC.

## INTRODUCTION

One of the stressors that livestock animals receive is owing to transport. Transport of livestock may expose animals to physical stimuli such as altered external conditions or changes in temperature, physiological stimuli such as restriction of feed and water during transport, and psychological stimuli due to exposure to new environments [1]. Changes in the environment during transport stimulate emotions and lead to physical fatigue, resulting in physiological changes in livestock. Thus, transport is considered to be a cause of discomfort and stress in livestock [2–4]. In addition, the lack of post-transit facilities and inadequate feed and water supply can also lead to post-transport stress [5]. These stresses have negative consequences for the health of livestock such as changes in biochemical function, endocrine balance, and pathological variables, and can sometimes even lead to death [6,7]. The stress response consists of the action of the hypothalamic-sympathetic nervous system and the hypothalamic-pituitary-adrenal (HPA) axis. The hypothalamus-sympathetic nervous system recognizes external stressors via signals in the cerebral cortex and secretes catecholamines, such as epinephrine and norepinephrine, in the adrenal medulla and brain [8–10]. The action of HPA is mediated by corticotrophin-releasing factor produced by the paraventricular nucleus in the hypothalamus, which leads to the secretion of β-endorphin and adrenocorticotropic hormone (ACTH). This ACTH leads to secretion of glucocorticoids in the adrenal cortex, which has negative effects on livestock [9]. While research has been carried out on the physiological and behavioral changes in ruminant livestock owing to stress after transport, most of these studies have been carried out on young calves and cattle. In particular, there are few studies on the changes in physiology and productivity in milking cattle. Hence, the present study was conducted to investigate the physiological changes caused by transport stress and the changes in milk yield and milk composition of lactating dairy cows to provide fundamental data for improving the welfare of livestock and promoting milk production.

## MATERIALS AND METHODS

### Animals

Ten non-pregnant, clinically healthy Holstein lactating dairy cows (average age 41 months, 523.8 kg, 1.5 parity number, 123 days in milk, milk yield 34 kg) were used. The cows were provided free access to feed and water which were supplied at 10:00 AM daily. The feed used in this study was a total mixed ration (TMR) which was composed of alfalfa, ryegrass straw, cottonseed, beet pulp, and commercial concentrates.

The cows had no history of mastitis or peripartum disease and had no previous experience of road transport. This study conformed to the Guide for the Care and Use of Laboratory Animals published by the US National Institutes of Health (NIH publication No. 85-23, revised 1996, latest revision in 2011) and was approved by the Konkuk University Animal Welfare Committee (KU 09021).

### Transport

The cows were randomly divided into two groups—a transported group (TG, n = 5) and a non-transported group (NTG, n = 5). TG cows were loaded three and two heads separately, fixing heads with polypropylene rope of 8 mm diameter in two uncovered trucks (2.2×6.5 m, wood flooring) exclusively used for livestock transport. The trucks were equipped with iron guardrails and returned to the point of departure after transport for 4 h at an average speed of 50 km/h on an asphalt paved road. Blood and milk samples were collected from the cows in trucks stopped for 15 min during transport at 1, 2, and 4 h. Temperature and relative humidity during the transport period were average 3.3°C and 76.9%, respectively. No accidents occurred during the transport period. TG cows could be not fed TMR and water during transport, but all cows included in the NTG were confined to a pen where they were fed TMR and water after the 4 h transport. The NTG cows were housed in a pen with iron stanchions at the experimental farm as the points of departure and destination under the same feeding conditions as those for the TG cows.

### Sample collection

**Figure 1:**
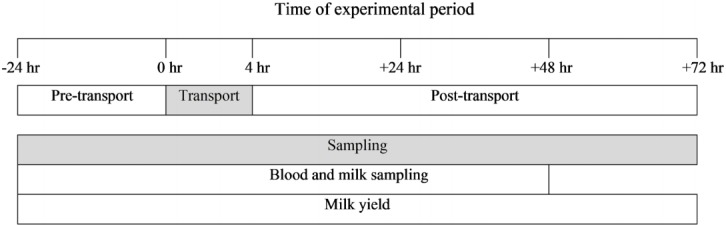
Schematic diagram of the experiment. Blood and milk samples were collected 7 times: pre-transport (−24 h), during transport (1, 2, 4 h), and post-transport (+2, +24, +48 h). Milk yields were measured every morning for 4 days.

Blood samples were collected via jugular venipuncture by a veterinarian. Two 3 mL blood samples of all cows were collected into evacuated tubes containing ethylenediaminetetraacetic acid K_2_ or clot activator; blood samples were collected pre-transport and during transport (−24, 0, 1, 2, and 4 h) and post-transport (+2, +24, and +48 h) from cows in trucks and individual pens, as shown in Figure 1. Next, milk samples were collected pre-transport and during transport (−24, 0, 1, 2, and 4 h) and post-transport (+2, +24, +48 h) from four teats using hands. Before the milk sample collection, the teat was washed once with a wet towel and the water was completely removed with a dry towel, and the teat was disinfected with 70% alcohol. And then the first milk was thrown out and milk samples were collected in more than 15 mL per teat using 50 mL conical tube. After mixing four samples, 15 mL of this mixture was collected to use as the milk sample for the assay. All samples were placed on ice and immediately transferred to the laboratory for analysis. Blood samples for cortisol measurement were centrifuged at 3,000×*g* for 20 min at 4°C to obtain the serum samples. The supernatants were stored in microtubes. All samples were immediately frozen at −80°C and stored at −80°C until analysis. Milk yields were measured every morning for 4 days using a milking equipment (Dematron 70, Westfaliasurge, Düsseldorf, Germany) as shown in Figure 1.

### Blood and milk analysis

The white blood cell count and number of neutrophil, lymphocyte, monocyte, eosinophil, basophil, and red blood cells in the blood samples were measured using an automatic analyzer (Hemavet 850FS, CDCtech, Oxford, CT, USA). Hemoglobin levels were measured using an automated coulter analyzer (STKS, Instrumentation Laboratories, Bedford, MA, USA). Concentrations of milk fat, protein, lactose, solids-not-fat, and milk urea nitrogen were measured using Milkoscan FT 6000 (Foss Electric A/S, Hiller, Denmark). Somatic cell count (SCC) was analyzed using Fossomatic 5000 counter (Foss Electric Co., Hillerød, Denmark).

### Cortisol measurements

Serum cortisol concentrations were determined using 1470 Wizard (Perkin Elmer, Turku, Finland) that uses the antibody-coated tube method of radioimmunoassay.

### Statistical analysis

The data in each group were statistically compared using T-TEST in Statistical Analysis System (SAS Institute, Cary, NC, USA, 2002). Significant differences between the TG and NTG were analyzed using Student t-test. Significant differences between pre-transport (−24 h) and during and post-transport (0, 1, 2, 4, + 2, +24, +48, +72 h) in both groups were analyzed using paired t-test.

## RESULTS

### Effects of transport stress on white blood cell and differential counts in lactating cows

**Figure 2:**
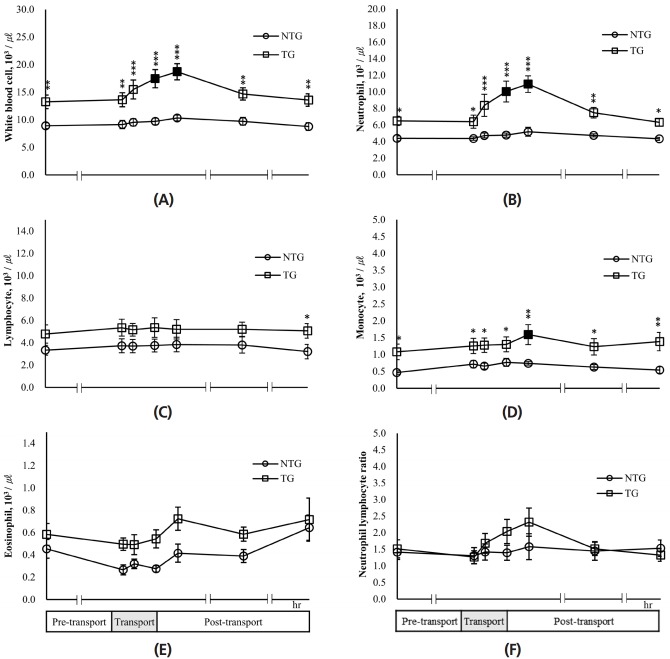
Effects of transport stress on white blood cell and differential counts in lactating cows. White blood cell count (A), numbers of neutrophils (B), lymphocytes (C), monocytes (D), and eosinophils (E), and neutrophil:lymphocyte ratio (F) were measured for the pre-transport (−24 h), during transport (1, 2, 4 h), and post-transport (+2, +24, +48 h). NTG, non-transported group; TG, transported group. ■ ●: Represent the significant difference (p<0.05) in the data after transport periods compared with pre-transport (−24 h). Asterisks mean significant difference between NTG and TG (* p<0.05, ** p<0.01, *** p<0.001).

As shown in Figure 2A, the white blood cell count of the TG was significantly (p<0.05) higher than those of the NTG for each experimental time (pre-transport, transport, and post-transport). In the TG, the white blood cell counts at 4 h transport and 2 h post-transport were 17.46±1.65 and 18.72±1.47×10^3^/μL, respectively. These values were significantly (p<0.05) higher by 31.7% and 41.2%, respectively, compared to those seen at 24 h pre-transport. The white blood cell count in the NTG increased during experiment compared to that observed for pre-transport; however, this increase was not significant. The neutrophil numbers of the TG were significantly higher than those of the NTG for each experimental time (Figure 2B). Additionally, the neutrophil numbers of the TG continuously increased until 2 h post-transport, and those for transport 4 h and post-transport 2 h were significantly (p<0.05) higher than those of pre-transport. In the NTG, the neutrophil number increased by 18%, while that in the TG increased by 68%.

On the other hand, the lymphocyte number of the TG were higher than those of the NTG for each experimental time except 48 h post-transport, and there was no significant difference between groups (Figure 2C). However, the lymphocyte number of the TG (5.07±0.65×10^3^/μL) significantly (p<0.05) increased compared with that of the NTG (3.22±0.65×10^3^/μL) at 48 h post-transport. Both groups showed no change in lymphocyte numbers during the experimental period compared to that seen at 24 h pre-transport, respectively. The monocyte numbers in the TG were significantly (p<0.05) higher than those in the NTG for each experimental time (pre-transport; −24 h, transport; 1, 2, 4 h, and post-transport; 2, 24, 48 h) (Figure 2D). In the TG, monocyte numbers at 2 h post-transport (1.59±0.29×10^3^/μL) were significantly (p< 0.05) higher than those seen at 24 h pre-transport (1.08±0.23 ×10^3^/μL). The number of eosinophils in the TG were similar to those of the NTG for each experimental time (Figure 2E). Neutrophil lymphocyte ratios of the TG and NTG were 1.27 to 2.32 and there was no significant difference between the two groups and between 24 h pre-transport and each experimental time (transport 1, 2, 4 h and post-transport 2, 24, 48 h) in each group (Figure 2F). However, neutrophil–lymphocyte ratios of the TG were 45% and 46% higher than those of the NTG at the end of transport (4 h) and 2 h post-transport, respectively.

### Effects of transport stress on blood parameters in lactating cows

**Figure 3:**
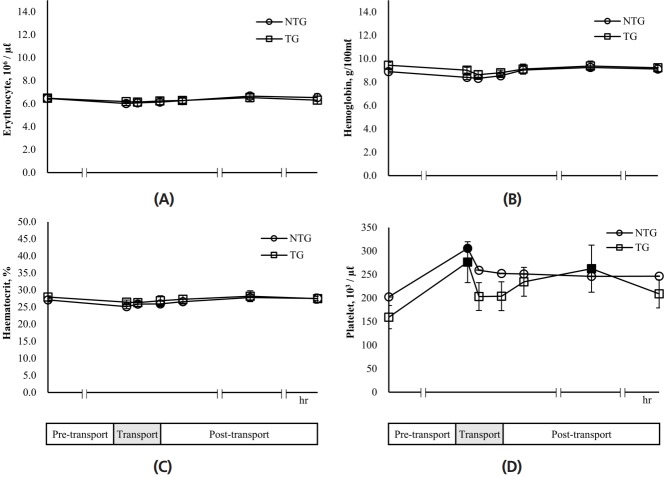
Effects of transport stress on blood parameters in lactating cows. Erythrocyte (A), hemoglobin (B), hematocrit (C), and platelet (D) were measured for the pre-transport (−24 h), transport (1, 2, 4 h), and post-transport (+2, +24, +48 h). NTG, non-transported group; TG, transported group. ■ ●: Represent the significant difference (p<0.05) in the data after transport periods compared with pre-transport (−24 h).

As shown in Figure 3A, the erythrocyte numbers in the NTG and TG were 6.02 to 6.66×10^6^/μL and 6.15 to 6.53×10^6^/μL, respectively during the experiment. There was no significant difference between the two groups and between 24 h pre-transport and each experimental time (transport 1, 2, 4 h and post-transport 2, 24, 48 h) in each group. The levels of hemoglobin in the TG (8.64 to 9.44 g/100 mL) were a little higher than those in the NTG (8.32 to 9.26 g/100 mL) at each experimental time (Figure 3B). The hemoglobin levels in the NTG and TG were 8.32±0.09 g/100 mL and 8.64±0.32 g/100 mL at 2 h transport, respectively, which were the lowest in the experimental period. However, hemoglobin levels recovered to those of 24 h pre-transport in both groups after the end of transport. The hematocrit values of the TG were similar those of the NTG at each experimental time (Figure 3C). Furthermore, there was no difference in these values between 24 h pre-transport and each experimental time (transport 1, 2, 4 h and post-transport 2, 24, 48 h) in each group. In the NTG, the platelet number dramatically increased at 1 h transport, showing a significant (p<0.05) difference compared to that seen at 24 h pre-transport: 305.60±43.53×10^3^/μL for the transport 1 h and 202.60±28.27×10^3^/μL for the pre-transport 24 h (Figure 3D). This number decreased after 2 h transport and was maintained at 246.40 to 259.00×10^3^/μL over time. Also, the platelet number in the TG was 276.40±43.45×10^3^/μL at 1 h transport, which was significantly (p<0.05) approximately 73% higher than that at 24 h pre-transport. After 2 h transport, the platelet number in the TG decreased but then increased significantly (p<0.05) to about 64% compared with that of 24 h pre-transport. However, there was no difference between the TG and NTG at each experimental time.

### Effects of transport stress on serum cortisol in lactating cows

**Figure 4:**
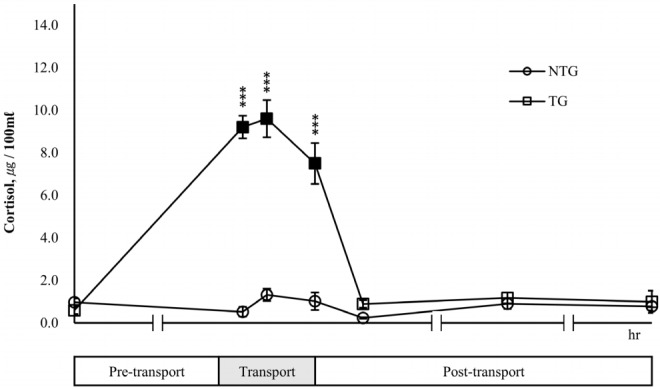
Effects of transport stress on serum cortisol in lactating cows. Serum cortisol was measured for the pre-transport (−24 h), transport (1, 2, 4 h), and post-transport (+2, +24, +48 h). NTG, non-transported group; TG, transported group. ■ ● : Represent the significant difference (p<0.05) in the data after transport periods compared with pre-transport (−24 h). Asterisks mean significant difference between NTG and TG (* p<0.05, ** p<0.01, *** p<0.001).

In the TG, a significant (p<0.05) increase in cortisol concentration was observed during transport and the maximum values were recorded at 2 h transport (24 h pre-transport vs 2 h transport = 0.59 μg/100 mL vs 9.61 μg/100 mL) (Figure 4). However, the cortisol levels of the TG decreased to 0.89 μg/100 mL after 2 h post-transport and then recovering to the level of 24 h pre-transport. While, there was a slight change in the cortisol concentration of the NTG, there was no significant difference compared to the cortisol levels seen for 24 h pre-transport. During transport, cortisol levels of the TG were 7.50 to 9.61 μg/100 mL, which were significantly (p<0.05) higher than those of NTG with 0.53 to 1.32 μg/100 mL.

### Effects of transport stress on daily milk yields and milk composition in lactating cows

**Figure 5:**
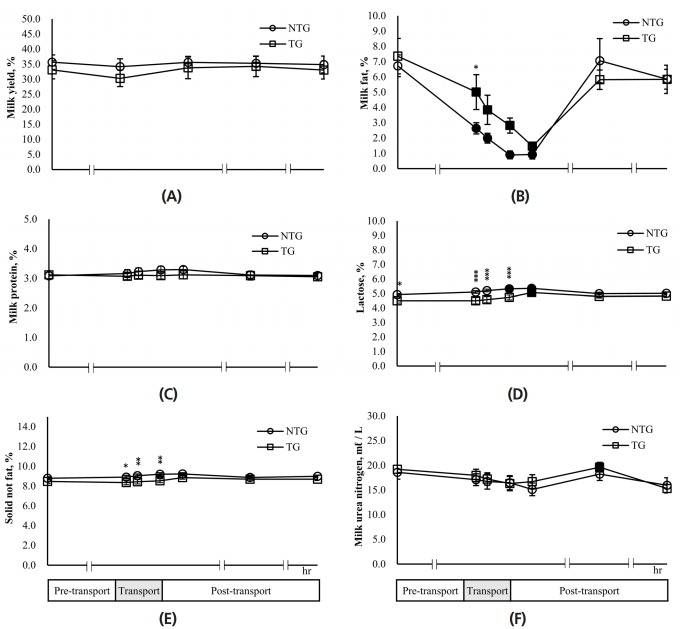
Effects of transport stress on daily milk yields and milk composition in lactating cows. Milk yield (A) was measured for the pre-transport (−24 h), transport (0 h), and post-transport (+24, +48, +72 h). Milk fat (B), protein (C), lactose (D), solid-not-fat (E), and milk urea nitrogen (F) were measured for the pre-transport (−24 h), transport (1, 2, 4 h), and post-transport (+2, +24, +48 h). NTG, non-transported group; TG, transported group. ■ ●: Represent the significant difference (p<0.05) in the data after transport periods compared with pre-transport (−24 h). Asterisks mean significant difference between NTG and TG (*p<0.05, ** p<0.01, *** p<0.001).

Milk yields were measured at 24 h pre-transport, 0 h transport, 24 h, 48 h, and 72 h, post-transport, respectively (Figure 5A). Milk production in the NTG and TG remained relatively constant during the experiment, but milk yields of the TG were less than those of the NTG. Milk yield of the TG was 30.32± 3.62 kg/d at 0 h transport and a little decreased in comparison with the 24 h pre-transport. However, this recovered to the level of the 24 h pre-transport after 24 h post-transport, i.e., 32.92±3.01 kg/d and maintained to 33.82 to 34.30 kg/d. To measure the change in milk components due to transport, milk was collected at pre-transport, 1, 2, 4 h transport, and 2, 4, 24, 48, and 72 h post-transport. During transport and at 2 h post-transport, the milk fat content of the NTG significantly (p<0.05) decreased by 60% to 86% compared to the 24 h pre-transport, but recovered to the level of 24 h pre-transport after the 24 h post-transport (Figure 5B). The milk fat content of the TG significantly (p<0.05) decreased by 32% to 80% compared to that observed for 24 h pre-transport during the same period and recovered to the level seen for 24 h pre-transport after 24 h post-transport. On the other hand, the milk fat content in the TG were higher than those in the NTG during transport and at 2 h post-transport. In particular, the level of milk fat in the TG at the 1 h transport was significantly (p< 0.05) 1.9 times higher than that in the NTG. The milk protein levels in the NTG were 3.0% to 3.3% and those in the TG were 3.0% to 3.1%; there was no significant difference between two groups and between 24 h pre-transport and each experimental time (transport 1, 2, 4 h and post-transport 2, 24, 48 h) in each group (Figure 5C). The lactose content in the NTG was 4.93%±0.06% at the 24 h pre-transport and increased with the initiation of transport (Figure 5D). The lactose content recovered to 4.99%±0.08% at 24 h post-transport. Especially, the lactose content at 4 h transport and 2 h post-transport were 5.32%± 0.09% and 5.35%±0.08%, respectively, which were significantly (p<0.05) higher than those of 24 h pre-transport. The lactose levels in the TG showed a similar tendency to those in the NTG. Lactose content in the TG was 5.07%±0.13% at 24 h post-transport, which significantly (p<0.05) increased by 12% compared to that of 24 h pre-transport. Overall, the lactose content of the TG was a little lower than those of the NTG at each experimental time. However, lactose levels of the TG were significantly (p<0.05) lower than those of the NTG at 24 h pre-transport and during transport. During transport (1, 2, 4 h), solid not fat content of the TG and NTG showed 8.36% to 8.55% and 8.92% to 9.20%, respectively, with levels in the TG being significantly (p<0.05) lower than those in the NTG (Figure 5E). Furthermore, the levels in the TG and NTG showed no significant difference between the pre-transport 24 h and each experimental time (transport 1, 2, 4 h and post-transport 2, 24, 48 h) in each group. Milk urea nitrogen showed no difference between the NTG and TG at each experimental time (Figure 5F). During transport and at 2 h post-transport, milk urea nitrogen content slightly reduced compared to those of 24 h pre-transport in both groups, respectively and recovered to the level seen for 24 h pre-transport and 24 h post-transport. However, those of the NTG and TG decreased again at 48 h and 72 h post-transport, respectively. Particularly, milk urea nitrogen of TG at 48 h post-transport was significantly (p<0.05) reduced by approximately 20% compared to 24 h pre-transport.

### Effects of transport stress on milk somatic cell count

During transport and at 2 h post-transport, the SCC of the NTG tended to reduce but sharply increased at 24 h post-transport (Table 1). Further, these recovered to the levels observed 24 h pre-transport at 48 h post-transport. However, the SCC of the TG was significantly (p<0.05) higher at 1 h during transport by about 3.6 times than that at 24 h pre-transport; this then reduced to 128.4 to 173.4×10^3^/mL after the end of transport (from 2 h post-transport to 48 h post-transport).

## DISCUSSION

The present study was performed to investigate the effects of transport stress on physiological and hematological responses and milk performance in lactating dairy cows. Our results demonstrated that transport stress lead to increased leucocyte, neutrophil, and lymphocyte numbers as a result of elevated cortisol levels in lactating cows.

Transport stress is known to lead to increased total leucocyte count and neutrophil, eosinophil, and monocyte count in livestock [11]. In the present study, the total leucocyte, neutrophil, and monocyte numbers in lactating cows transported over 200 km for 4 h were significantly higher than those of the NTG at each experimental time. Leukocytes and neutrophil counts of the TG were high both during transport 2 h post-transport. Yagi et al [12] have reported that the increase in leukocyte numbers is due to an increase in neutrophil numbers, which together constitute a high proportion of white blood cells. In addition, glucocorticoids are known to stimulate the inflow of neutrophils into the bone marrow and blood vessels and inhibit their migration to other tissues [13]. Based on the results of the present study, transport stress appears to lead to a 13 to 16-fold higher cortisol concentration than that before transport, resulting in the increase in the number of neutrophils. Seixas et al [14] reported that heat stress promoted the secretion of stress hormones, which led to increased number of leukocytes in Brazilian hair sheep. Our results resemble those of Tarrant et al [15], who he observed that total leukocyte and neutrophils were higher by 23% to 68% in Friesian steers after transporting 1,000 km by an articulated truck for 24 h than in those before transport. On the other hand, transport did not affect eosinophil numbers in the present study. However, Ali-Gholi et al [16] reported that the number of eosinophils was decreased 3 h post-transport in hybrid dairy cows transported by truck for a 40 km round trip. This disparity in the results might be due to differences in transport distance and time.

The neutrophil:lymphocyte ratio is used as a general measure of response to stress. Stress results in a decrease in the number of lymphocytes and an increase in that of neutrophils, leading to an increase in the neutrophil:lymphocyte ratio [17]. Based on the results of the present study, there was little change in the neutrophil:lymphocyte ratio in non-transported cows during the experiment, whereas in transported cows, this ratio was higher by 34% to 50% at the end of transport and 2 h post-transport than that at 24 h pre-transport, because neutrophils were greatly increased in the same time period. Trucking for 4 h appears to be stressful to lactating cows. However, the red blood cell count, hemoglobin levels, and hematocrit were not influenced by transport. These results were consistent with those of a study on wild goats transported for 100 minutes [18] and that on Friesian steers transported for 3 h [19]. In contrast, Earley et al [20] reported that the red blood cell count, hemoglobin level, and hematocrit in Holstein–Friesian bull calves were significantly higher after transporting them for 474 km by an articulated truck for 8 hours. There was also a significant increase in hematocrit in Holstein steers subjected to 15 h transport stress [21]. These results were not consistent with our observations. Several experiments have suggested that long-term road transport induces dehydration, leading to decreased plasma volume [19,21–23], and the activation of the sympathetic nervous system by transport stress leads to the contraction of the spleen, causing the red blood cells in the spleen to flow into the blood, thereby increasing the erythrocytes and hematocrit [24,25]. The spleen is well known to be an erythrocyte reservoir [26]. In addition, erythropoiesis is regulated by a well-coordinated mechanism to maintain the normal range of red blood cells despite changes in environmental conditions [26]. Therefore, hematocrit appears to be influenced by transport time. However, hematocrit levels may have been maintained during transport in the present study because 4 h of transport in was probably not sufficient to affect the number of erythrocytes and the spleen in dairy cows. Exposure to a new environment or stress caused by handling or transport affects HPA and promotes the release of glucocorticoids from the adrenal cortex in livestock [27]. In general, the levels of cortisol and catecholamine, a type of glucocorticoids, are widely used as indicators of stress [27,28]. In the present study, cortisol secretion was increased sharply as a result of transport stress after the start of transport, and was about 13 to 16 times higher than that 24 h pre-transport. Cortisol concentration began to decrease after the end of transport and almost recovered to the pre-transport level. These results are consistent with those of Yagi et al [12], who observed that cortisol level peaked at 2 h during transport and recovered to the pre-transport level at 2 h post-transport in dairy cows transported for 4 h, and that no change in cortisol concentration was observed in the control group, which was not transported [29]. Similar results have also been obtained in experiments conducted on sheep [30], goats [31,32] and calves [33].

Changes in daily milk yield were investigated once a day from 1 day pre-transport to 3 days post-transport. Milk production of the TG with increased SCC was a little lower than that of the NTG. According to Cinar et al [34], milk SCC affects milk production—milk yield is reduced by increased SCC. This finding was consistent with previously reported results that heat stress decreases milk yield in lactating cows [35,36]. Meanwhile, the milk SCC of the TG increased sharply during transport and was higher than that in dairy cows restrained by stanchion throughout the experiment in the present study. These results are similar to those of a previous report stating that SCC was increased when 9 lactating cows were transported for 4 h [12]. SCC is also the most commonly used indicator of milk quality [37,38]. The milk quality of the TG was lowered owing to the elevated SCC caused by transport stress. Milk somatic cells include 75% leucocytes and 25% epithelial cells [37]. Therefore, the elevated SCC during transport seems to be influenced by the significant increase in leucocyte number owing to transport stress. After transport, the SCC of the TG decreased to 128 to 174×10^3^/mL and milk quality improved in a manner similar to that in the NTG. Generally, milk quality is held to be good when milk SCC is less than 200×10^3^/mL [37,38]. In addition, the elevated SCC negatively affected milk constituents because SCC is related to milk quality [34,37]. Protein and solid non-fat levels were not changed during transport in both groups but protein, solid non-fat, and lactose content in the TG was lower than that in the NTG throughout the experiment, and the lactose levels in the TG and NTG increased with the start of transport and restraint, respectively. Lactose levels in the NTG recovered to the original level at 24 h post-transport, whereas lactose levels in the TG recovered at only 72 h post-transport. However, the milk fat content in the TG was higher than that in the NTG, and milk fat levels of both groups decreased over 4 h of transport and restraint, respectively. According to Harmon [39], the increased SCC reduces the lactose and fat content of milk by reducing the synthetic activity of the mammary tissue. Some studies, however, have reported no change in fat content [40–42]. The results of this experiment are considered to be accounted for as follows: the decreased fat content of milk is due to the variation among lactating cows rather than the effects on mammary tissue due to elevated SCC.

Taken together, we observed that 4-h transport led to increased plasma cortisol, which was responsible for the effects on some blood parameters—transport stress increased leucocyte, neutrophil, eosinophil, and monocyte numbers. In addition, transport stress may bring about increased milk SCC and decreased milk yield in lactating dairy cows. Therefore, further studies are required to identify measures to mitigate these changes caused by transport stress to improve well-being, health, milk production, and milk quality in lactating dairy cows.

## Figures and Tables

**Table 1:** Changes in somatic cell count of lactating cows pre-, during and post-transport

Time	NTG	p value1)	TG	p value1)	p value2)
Somatic cell count, ×10^3^/mL					
−24	76.80±25.21	-	184.00±48.15	-	0.5718
1	68.40±18.10	0.9646	677.20±420.50	0.0107	0.0018
2	48.40±9.50	0.8808	438.20±228.18	0.1820	0.0422
4	26.20±4.68	0.7894	305.00±132.50	0.5235	0.1437
+2	33.60±8.52	0.8196	128.40±65.80	0.7692	0.6170
+24	161.40±67.31	0.6553	173.40±43.50	0.9554	0.9495
+48	78.20±19.55	0.9941	138.20±41.57	0.8090	0.7515

1) Pre-transport time vs during transport or post-transport time.

2) NTG (non-transported group) vs TG (transported group).

Data are represented as mean±standard error.
